# Immune and non-immune hydrops fetalis in a resource-limited setting: a report of two cases

**DOI:** 10.11604/pamj.2026.53.141.52080

**Published:** 2026-03-24

**Authors:** Willis Okumu Ochieng, Rosa Ndiema Chemwey, Moses Kipchumba Lagat, Elijah Kagondu Thagana

**Affiliations:** 1 Department of Obstetrics and Gynaecology, University of Nairobi, Nairobi, Kenya,; 2 Department of Obstetrics and Gynaecology, Kenyatta National Hospital, Nairobi, Kenya

**Keywords:** Hydrops fetalis, immune hydrops fetalis, non-immune hydrops fetalis, intrauterine transfusion, case report

## Abstract

Hydrops fetalis is a fetal condition characterized by abnormal fluid accumulation in multiple fetal compartments and is associated with high perinatal mortality. Unlike high-income settings, both immune and non-immune hydrops fetalis (IHF and NIHF) remain relevant in low- and middle-income countries (LMICs), where evaluation and specialized care are often delayed or inaccessible. We report two cases of hydrops fetalis diagnosed antenatally at a tertiary referral hospital in Kenya, followed through delivery and outcome. Case one involved immune hydrops fetalis due to suspected non-Rhesus alloimmunization, presenting with fetal ascites and cardiac dysfunction. Management included paracentesis and intrauterine transfusions, which temporarily stabilized the fetus, but the neonate died on day two of life. Case two involved non-immune hydrops fetalis initially presented with isolated pleural effusion, which progressed to generalized hydrops. Amniotic fluid testing showed Rubella IgM positivity. Management included thoracocentesis and conservative management till 34+0 weeks´ gestation. Unfortunately, the pregnancy resulted in an intrauterine fetal demise. These cases illustrate the wide spectrum of hydrops fetalis and the diagnostic and therapeutic challenges in resource-limited settings. While antenatal interventions may prolong gestation, outcomes are largely determined by the underlying cause and disease severity.

## Introduction

Hydrops fetalis is described as excessive, abnormal extracellular fluid accumulation within two or more tissues or body cavities, including the pericardial, pleural, or peritoneal spaces, and skin. Other findings include polyhydramnios and placental thickening. These findings are detected by ultrasound. Several underlying etiologies can result in the development of hydrops fetalis, which include both immune and nonimmune-mediated processes. The overall perinatal outcomes are poor [1-5]. The incidence of maternal rhesus alloimmunization leading to fetal immune hydrops is declining in high-income settings due to treatment with anti-D immunoglobulin. However, it has remained a significant cause of hydrops fetalis in areas of the world where treatment is less accessible. Alloimmunization due to other red cell antigens is also possible. Ninety percent of cases of hydrops fetalis, therefore, have a non-immune etiology, which includes chromosomal, cardiovascular, fetal infections, haematologic, fetal/placental tumours, and twin-to-twin transfusion syndrome. The final common pathway in hydrops fetalis is impaired fluid regulation processes due to decreased plasma oncotic pressure and lymphatic flow, and increased hydrostatic capillary pressure and endothelial damage [1-3,5]. Whether red cell alloimmunization is present or not is a crucial step in the evaluation of hydrops fetalis. To identify cardiac abnormalities and fetal anemia, respectively, fetal echocardiography and Doppler examination of the middle cerebral artery peak systolic velocity (MCA-PSV) are crucial. The MCA-PSV, expressed in multiples of the median (MoM), is used to determine the appropriate timing for intrauterine transfusion. Maternal serologic evaluation for infectious etiologies such as cytomegalovirus (CMV), toxoplasmosis, syphilis, and parvovirus B19 is likewise recommended [2,6-10].

When a non-immune cause is established, comprehensive imaging with detailed ultrasonography or magnetic resonance imaging should be performed to detect associated structural anomalies. Additional genetic tests, such as chromosomal microarray analysis, karyotyping, and targeted gene testing, may be required if structural abnormalities are found. In low-resource settings, however, detailed evaluation is limited by the affordability and availability of these tests [2,5]. The management of hydrops fetalis depends on the etiology and gestational age. Intrauterine intravascular transfusions are performed between 19 and 34 weeks of gestation. Prior to 18 weeks, fetal transfusions are rarely successful due to the low caliber of vessels hindering intravascular access, intraperitoneal transfusion is performed. Antenatal corticosteroids are given due to the risk of preterm delivery, and depending on where fluid has accumulated, fluid diversion procedures such as pericardiocentesis, paracentesis, and thoracocentesis may relieve compression and improve perinatal outcomes. Treatable fetal infections or cardiac conditions are associated with better outcomes [2,7]. In LMICs, running all the recommended tests for hydrops fetalis is hindered by financial difficulties and limited resources to perform the tests. Additionally, data from LMICs is scarce due to incomplete evaluation of cases owing to financial limitations [4,5]. We report two cases of hydrops fetalis to highlight the differing etiologies, clinical presentations, including differing patterns of fluid accumulation, antenatal interventions, response to treatment, and outcomes in a resource-limited setting.

## Patient and observation

### Case one: immune hydrops fetalis

**Patient information:** a 32-year-old primiparous woman at 29+1 weeks of gestation was referred for maternal-fetal surveillance following routine prenatal ultrasonography that showed massive fetal ascites. Her obstetric history included one prior cesarean delivery resulting in a live neonate, and no prior history of hydrops fetalis or alloimmunization. She had no history of rubella or CMV infection or vaccination. Both the patient´s and the partner´s blood groups were O Rh-D positive.

**Clinical findings:** during presentation, the patient was clinically stable with normal vital signs. The fundal height corresponded with the gestational age. An ultrasound done at 27+5 weeks of gestation showed a viable singleton gestation with preserved cardiac activity and no structural anomalies, although massive ascites of approximately 545.62 mL was noted. Later, an ultrasound with fetal echocardiography was done at 30+0 weeks of gestation, which revealed cardiomegaly, pericardial effusion, and ventricular wall thinning. The middle cerebral artery peak systolic velocity (MCA-PSV) was noted to be elevated at 52.93 cm/s, corresponding to 1.39 multiples of the median (MoM), consistent with mild fetal anemia [7].

**Figure 1:**
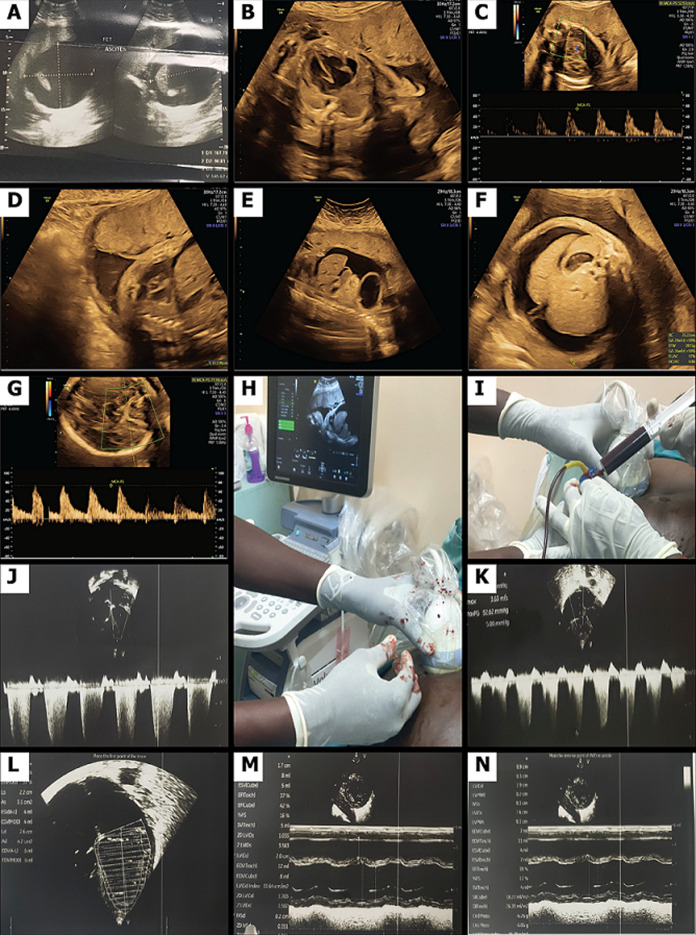
ultrasonographic findings and interventions across gestations, and postnatal echocardiographic findings (case one); A) massive fetal ascites at 27+5 weeks gestation; B-E) concurrent ultrasonography and fetal echocardiography at 30+0 weeks, showing cardiomegaly, pericardial effusion, thinning of the ventricular walls, and an MCA-PSV of 52.93 cm/s (1.39 MoM); F, G) progressive abdominal distension and skin edema consistent with worsening hydrops fetalis at 32+4 weeks; H, I) third ultrasound-guided intrauterine transfusion performed at 33+1 weeks; J, N) postnatal echocardiographic findings in the neonate

**Timeline:** Figure 1 shows the ultrasonographic findings and interventions across gestations, and postnatal echocardiographic findings. At 27+5 weeks, fetal ascites was noted on ultrasound. At 29+1 weeks, cordocentesis was performed with therapeutic fetal paracentesis, in which 440 mL of ascitic fluid was drained, followed by intrauterine transfusion (IUT). Pretransfusion fetal hemoglobin was 9.8g/dl, indicating moderate anemia. The fetal blood group was O Rh-D positive. A second IUT was performed at 30^+1^ weeks of gestation. At 32^+4^ weeks, another ultrasound was performed and showed partially resolved fetal ascites but new features of skin edema and abdominal wall thickening. Middle cerebral artery peak systolic velocity (MCA-PSV) had increased to 71.93 cm/s (1.6 MoM), indicating persistent fetal anemia. As a result, a third transfusion was done at 33+1 weeks. At 33+6 weeks, spontaneous preterm labour occurred, which necessitated an emergency caesarean delivery.

**Diagnostic assessment:** the patient's blood group was O-Rh-D positive with a positive indirect Coombs test. Extended red cell antibody testing was not available. Screening for HIV, syphilis, and hepatitis B was negative. Serial Doppler studies showed persistently elevated MCA-PSV values consistent with ongoing fetal anemia. Postnatal laboratory investigations revealed hypoproteinemia, hypoalbuminemia, mild anemia, thrombocytopenia, and impaired liver and renal function. Neonatal serologic testing demonstrated rubella and CMV IgG seropositivity. Neonatal echocardiography demonstrated dilated cardiomyopathy, moderate mitral and tricuspid regurgitation, persistent pulmonary hypertension, and a large patent ductus arteriosus with bidirectional shunting.

**Therapeutic intervention:** guided by the MCA-PSV/MoM, three ultrasound-guided intrauterine transfusions were performed at 29+1, 30+1, and 33+1 weeks of gestation using leukoreduced packed red blood cells. Therapeutic drainage of the fetal ascites was done during the initial cordocentesis to relieve the abdominal distension.

**Follow-up and outcome:** a live neonate with a poor Apgar score was delivered at 33+6 weeks. The neonate was intubated and put on mechanical ventilation. Paracentesis was done postnatally, and 80 mL of ascitic fluid was drained. Unfortunately, the neonate succumbed on day 2 of life.

**Patient perspective:** when she was told that her unborn baby had severe anemia and fluid in the body, she felt scared and overwhelmed. Each transfusion gave her some hope, but she couldn´t stop worrying. The team managing her explained things clearly, which helped her feel a bit more at ease. She was heartbroken after her baby died. She said that she hopes that in the future she will get early counseling and frequent check-ups to protect any future pregnancies.

**Case two: non-immune hydrops fetalis**

**Patient information:** a 25-year-old primiparous woman at 27+5 weeks of gestation was referred for maternal-fetal surveillance following routine prenatal ultrasonography that showed a significant isolated fetal pleural effusion. Her obstetric history included one prior cesarean delivery resulting in a live, preterm neonate, who died shortly after birth due to respiratory distress syndrome. She had no history of rubella or CMV infection or vaccination. The patient's blood group was O Rh-D positive.

**Clinical findings:** at presentation, the patient was clinically stable with normal vital signs. The fundal height corresponded with the gestational age, and the fetal heart rate was 157 bpm. An ultrasound done at 27+3 weeks of gestation showed a viable singleton gestation with a pleural effusion of approximately 26 mL. Later, an ultrasound done at 27+5 weeks of gestation showed that the hydrothorax had progressed, causing a mediastinal shift and compression of the lung. Doppler studies were normal.

**Figure 2:**
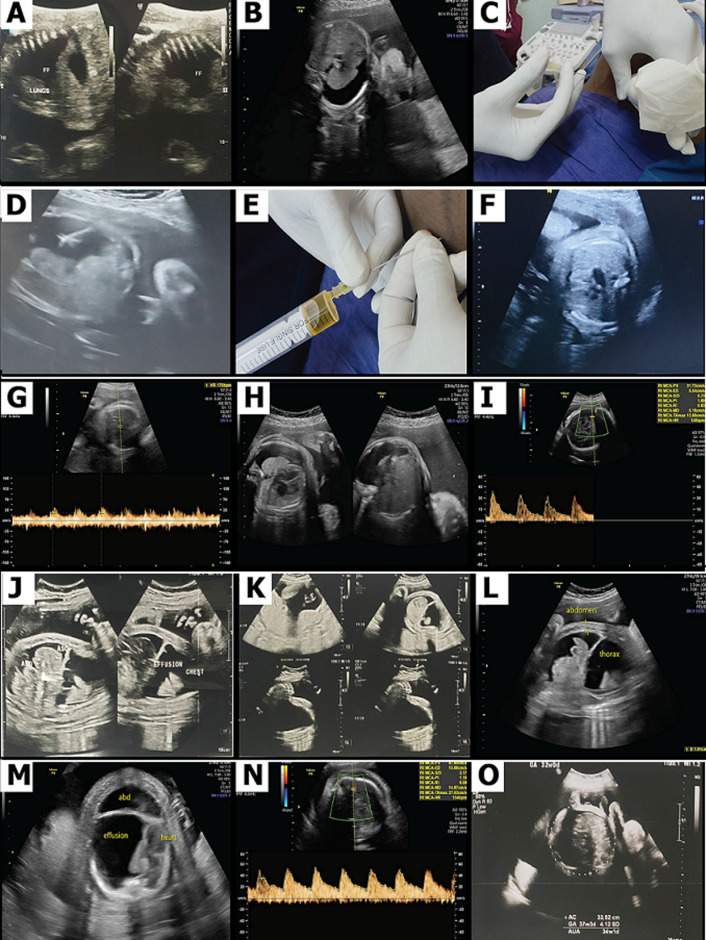
ultrasonographic findings and interventions across gestations (case two); A, B) isolated fetal pleural effusion at 27+3 weeks gestation; C-G) ultrasound-guided thoracocentesis performed at 27+5 weeks, with post-procedure lung re-expansion and confirmation of fetal viability; H, I) recurrent pleural effusion on follow-up imaging at 29+1 weeks; J-O) progression to generalized hydrops fetalis at 32+1 weeks

**Timeline:** Figure 2 shows the ultrasonographic findings and interventions across gestations. At 27+3 weeks, fetal hydrothorax was noted on ultrasound. At 27+5 weeks, thoracocentesis to decompress the lung was performed, in which 73 mL of pleural fluid was drained, and lung expansion was achieved. At 29+1 weeks, another ultrasound done showed that the pleural effusion had reaccumulated and was also compressing the fetal heart. A decision to manage the patient conservatively with an aim for delivery at ≤ 34+0 weeks was made. At 32+1 weeks, a follow-up ultrasound showed generalized hydrops fetalis (abdominal wall thickening, bilateral pleural effusion, ascites, and diffuse subcutaneous edema). Unfortunately, intrauterine fetal demise occurred at 32+6 weeks, and delivery was achieved through hysterotomy.

**Diagnostic assessment:** maternal screening for HIV, syphilis, and hepatitis B returned negative results. IgG was positive for both rubella and CMV in both maternal blood and fetal pleural aspirate. Subsequent testing of the amniotic fluid revealed rubella IgM positivity. The maternal indirect Coombs test was negative, suggesting a non-immune etiology. The progression from isolated pleural effusion to generalized hydrops was shown by serial ultrasonography. The MCA-PSV remained normal, supporting a non-anemic cause.

**Therapeutic intervention:** fetal lung decompression was performed at 27+5 weeks. Following recurrence of effusions, conservative management with intensive ultrasound surveillance was maintained, with a plan to prolong pregnancy to at least 34+0 weeks of gestation if feasible.

**Follow-up and outcome:** despite intervention and close monitoring, hydrothorax recurred and progressed to generalized hydrops fetalis. Intrauterine fetal demise at 32+6 weeks. Delivery was achieved via hysterotomy. The patient was advised to receive the rubella vaccine once she was medically stable and to delay conception for at least one month afterward, along with recommendations for preconception assessment and early antenatal care in future pregnancies. A comparative overview of both cases, including serial antenatal findings, interventions, and outcomes, is provided in Table 1.

**Table 1:** comparative summary of serial antenatal findings, interventions, and outcomes.

Timeline	Case 1: immune hydrops fetalis	Case 2: non-immune hydrops fetalis
**Serial antenatal ultrasound findings**		
Early third trimester	**27+5 weeks:** massive ascites (545.62 mL)	**27+3–5 weeks:** pleural effusion (26 mL) progressed to marked hydrothorax with mediastinal shift; Doppler preserved; MCA-PSV 28.3 cm/s
	**30+0 weeks:** hydrops, cardiomegaly, pericardial effusion, ventricular wall thinning; MCA-PSV 52.93 cm/s (1.39 mom)	**29+1 weeks:** hydrothorax re-accumulated; early cardiac strain
Mid third trimester	**32+4 weeks:** persistent ascites, resolution of pericardial effusion; MCA-PSV 71.93 cm/s (1.6 MoM)	**32+1 weeks:** generalized hydrops; massive pleural and intraperitoneal effusions
**Fetal Interventions**		
Early third trimester	**29+1 weeks**	**27+5 weeks**
	**Cordocentesis:** fetal Hb 9.8 g/dl; fetal blood group: O RhD positive	
	**Paracentesis:** 440 mL exudative fluid (protein 14.0 g/L)	**Thoracocentesis:** 73 mL (transudate)
	First intrauterine transfusion (42 mL PRBCs)	
	**30+1 weeks**	**Pleural fluid and maternal blood:** IgG positivity for CMV and rubella
	Second intrauterine transfusion (40 mL PRBCs)	
Mid third trimester	**33+1 weeks**	**32+1 weeks**
	Third intrauterine transfusion (120 mL PRBCs)	Amniocentesis: reactive for rubella IgM
	Donor blood characteristics: O negative, leukoreduced, <7 days old; hematocrit 61.6%, hemoglobin 20 g/dl	**Maternal indirect Coombs test:** negative
**Delivery & Outcome**		
Mid third trimester	**33+6 weeks:** delivered via cesarean section to a live neonate	**32+6 weeks:** delivered via cesarean hysterotomy; macerated stillbirth
	**APGAR**: 3 (1 minute), 5 (5 minutes), 6 (10 minutes)	
	Succumbed on day 2 of life	
Postnatal evaluation	CMV and rubella IgG positive	-
	**Multisystem dysfunction:** hypoproteinemia, hypoalbuminemia, mild anemia, thrombocytopenia, impaired hepatic and renal function)	
	**Echocardiography:** dilated cardiomyopathy, valvular regurgitation, pulmonary hypertension, patent ductus arteriosus.	

**Patient's perspective:** given her history of neonatal loss, the patient was apprehensive and upset upon hearing of the fetal pleural effusion. Although timely measures and monitoring gave her comfort, the development of intrauterine fetal death and widespread hydrops was tragic. She said she appreciated the healthcare team's communication and assistance and that she would be prepared to abide by the advice to be vaccinated and receive early prenatal care in the future.

**Informed consent:** written informed consent was obtained from both patients, and all information was de-identified to maintain confidentiality. This report was approved by the Kenyatta National Hospital-University of Nairobi Ethics and Research Committee (KNH-UoN ERC).

## Discussion

In this report, we included two antenatal cases that fulfilled diagnostic ultrasound criteria for hydrops fetalis. Our aim was to present differing etiologies, clinical presentations including differing patterns of fluid accumulation, antenatal interventions, response to treatment, and outcomes of hydrops fetalis managed in a resource-limited setting. These differences highlight the heterogeneity and importance of individualized management of hydrops fetalis [1,2]. In case one, an immune hydrops due to maternal red cell alloimmunization presented as fetal ascites and cardiac dysfunction. Management was comprised of therapeutic paracentesis and serial MCA-PSV-guided intrauterine transfusions. Unfortunately, persistent hydrops and cardiomegaly progressively worsened, culminating in neonatal death. In case two, a non-immune hydrops initially presented as an isolated fetal hydrothorax and was managed by ultrasound-guided thoracocentesis to relieve mediastinal shift and lung compression. However, the fetus subsequently developed generalized hydrops, resulting in an intrauterine fetal demise. Previous studies have shown that intrauterine transfusion and fluid drainage may stabilize the fetus and prolong pregnancy. In our reports, antenatal transfusions and fluid drainage provided temporary improvement but did not change the perinatal outcomes. This highlights the limited ability to reverse established hydrops fetalis [2]. The underlying cause determines prognosis. Mortality rates of up to 92% have been reported in utero heart failure. Low Apgar scores, acidosis, and hypoalbuminemia, which are signs of severe illness, are also associated with increased mortality [2]. In case one, persistent hydrops and cardiomegaly progressively worsened, culminating in neonatal death, while in case two, the presence of fluid in multiple compartments and fetal edema has also been associated with poorer outcomes [2]. Immune hydrops fetalis due to maternal red cell alloimmunization is rare in high-income settings due to effective anti-D prophylaxis. Immune hydrops fetalis is still prevalent in LMICs. This is mainly due to delayed diagnosis and incomplete evaluation, as seen in case one, where extended antibody testing was unavailable. As illustrated by case two, congenital infections such as rubella and CMV continue to play a major role in NIHF [1-3,6-8].

## Conclusion

This report highlights the challenges in diagnosing and managing hydrops fetalis in resource-limited settings. It also illustrates the generally poor prognosis of hydrops despite antenatal interventions such as intrauterine transfusion, thoracocentesis, and paracentesis. Improving outcomes will require detailed early antenatal care, wider antibody screening, better infectious disease screening, and improved access to maternal-fetal medicine services.
